# Solitary Neurofibroma of the Frontal Sinus

**DOI:** 10.1155/2012/373808

**Published:** 2012-08-09

**Authors:** Sudhir B. Sharma, Paul Hong

**Affiliations:** ^1^Georgetown Public Hospital Corporation and Department of Surgery, University of Guyana, Georgetown, Guyana; ^2^IWK Health Centre and Division of Otolaryngology-Head & Neck Surgery, Department of Surgery, Dalhousie University, 5850/5980 University Avenue, P.O. Box 9700, Halifax, Canada NS B3K 6R8; ^3^School of Human Communication Disorders, Dalhousie University, Halifax, Canada NS B3H 4R2

## Abstract

Isolated or solitary neurofibromas of the paranasal sinuses are rare. Mostly, they involve the maxillary sinus and so far, a solitary neurofibroma of the frontal sinus has never been reported in the literature. We present a case of frontal sinus neurofibroma treated successfully with surgical excision. A 28-year-old male presented with forehead swelling and computed tomography images revealed a tumor involving the left frontal sinus. Histopathological examination showed the tumor to be a neurofibroma. We discuss the clinical and pathological characteristics of neurofibroma arising in the nasal cavity and paranasal sinuses.

## 1. Introduction

Neurofibromas are benign nerve sheath tumors that originate from the peripheral nervous system. More specifically, they arise from the nonmyelinating Schwann cells but are commonly referred to as schwannomas in the literature [1]. Generally, neurofibromas can be categorized into dermal and plexiform subtypes. The former subtype is usually associated with a lone peripheral nerve in the integumentary system, while plexiform tumors are associated with many nerve bundles and can originate internally. Rarely, the plexiform tumors can undergo malignant transformation [1].

Neurofibromas are usually found in individuals with neurofibromatosis, which is an autosomal dominant disease. There are two types of neurofibromatosis: type 1 (von Recklinghausen disease), which is more common, and type 2, which typically has a more severe course due to central nervous system tumors [2].

On occasion, an isolated neurofibroma can transpire without being associated with neurofibromatosis. Mostly, these solitary tumors tend to occur in the gastrointestinal system [3, 4], and very rarely they have been reported to occur in the paranasal sinuses.

In this report, we describe a case of a solitary neurofibroma arising from the frontal sinus. To the authors' knowledge, no such case has been published to date.

## 2. Case Report

**Figure 1:**
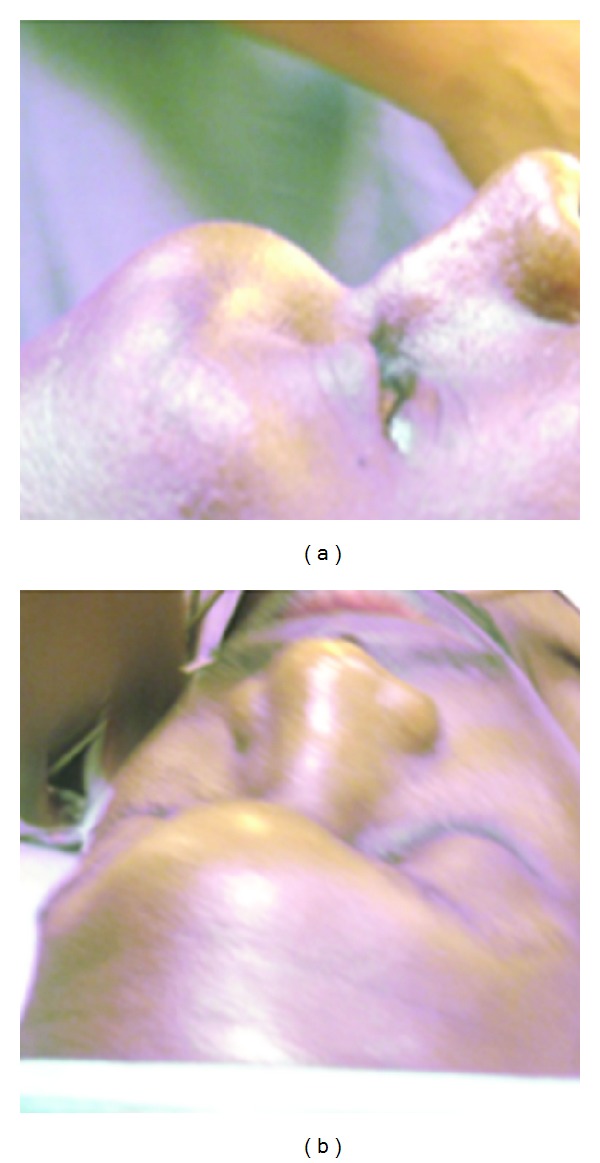
Photographs of the central forehead mass.

A 28-year-old man presented to the otolaryngology clinic with a central forehead swelling, which had been gradually enlarging over several months (Figure 1). There were no associated local regional signs or symptoms such as pain, headaches, epistaxis, nasal discharge or obstruction, and smell disturbances. No systemic symptoms, such as fever or weight loss, were present.

 Past medical history was unremarkable. There was no history of trauma or sinonasal disease. Social history was also unremarkable; the patient was a nonsmoker and an office worker with no prior exposure to toxic fumes or inhalants.

 On examination, there was a central forehead swelling just superior to the level of the medial eyebrows. The mass was estimated to be approximately 5 cm in size, oval shaped, and no overlying skin changes were noted (Figure 1). On palpation, the mass was firm and smooth; mild tenderness was elicited and no pulsations or bruits were observed. No other masses were detected and the intranasal examination did not reveal any other pathologies. Rest of the head and neck examination was normal.

**Figure 2:**
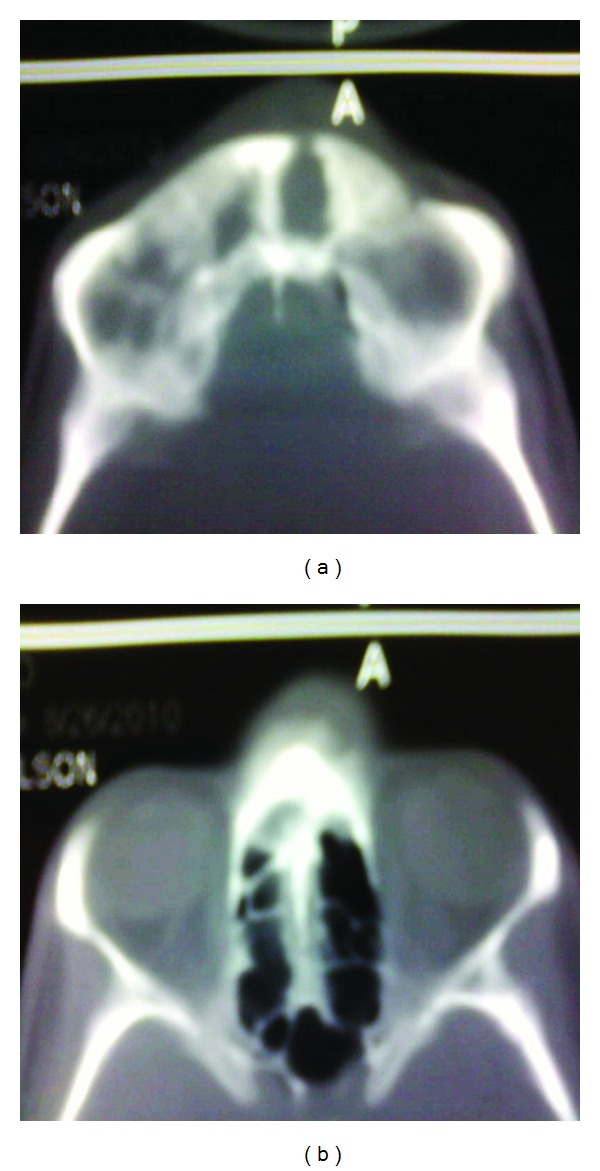
Axial CT scan images of the left frontal sinus tumor with anteroinferior frontal sinus wall erosion. The right frontal sinus contained purulent discharge (noted in the operating room).

 A computed tomography (CT) scan of the head and paranasal sinuses was obtained, which revealed an isolated soft tissue mass involving the left frontal sinus (Figure 2). The mass was found to have a locally expansile effect, with partial erosion of the anteroinferior frontal sinus wall. There was no extension into the nasal cavity or the ethmoid sinuses.

**Figure 3:**
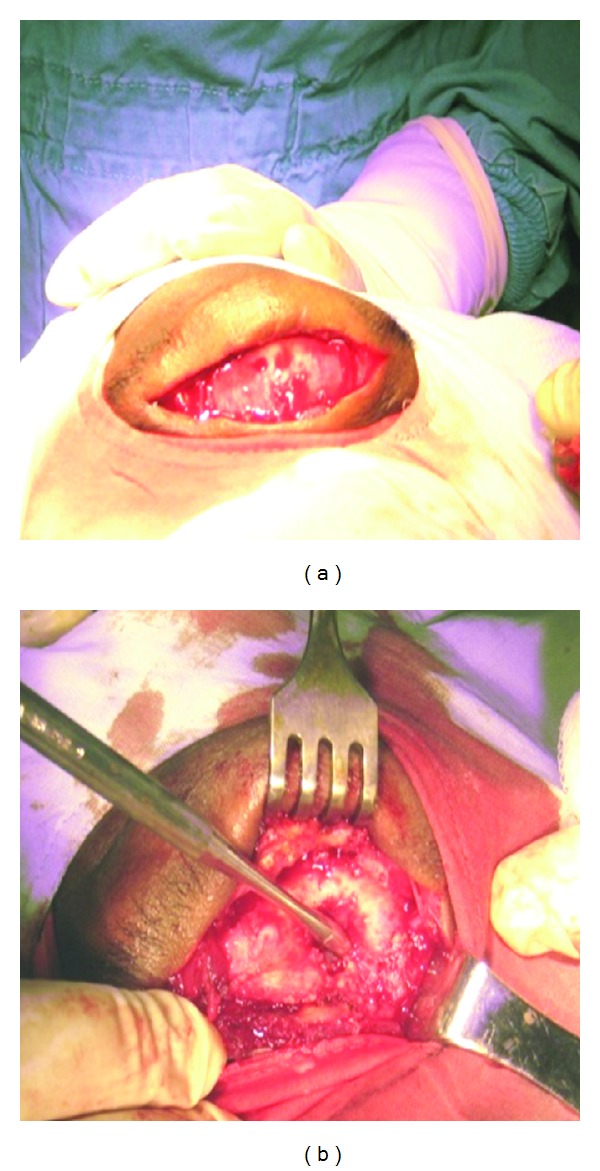
Intraoperative photographs on the frontal sinus tumor excision. (a) demonstrates the subcutaneous portion of the soft-tissue tumor, overlying the bony frontal sinus and forehead. (b) shows the frontal sinus bony defect after removal of the mass. (Note that the suction instrument placed through the bony dehiscence).

 On surgical exploration, the superficial component of the tumor, was found to be arising from a left frontal sinus bony dehiscence, which was approximately 1.5 cm in diameter located along the anteroinferior frontal sinus wall (Figure 3). The subcutaneous portion of the tumor was removed first, along with a margin of frontalis muscle, and the intrasinus component was excised through the bony dehiscence. (Please note that endoscopic sinus surgery equipment is not available at the lead author's institution).

**Figure 4:**
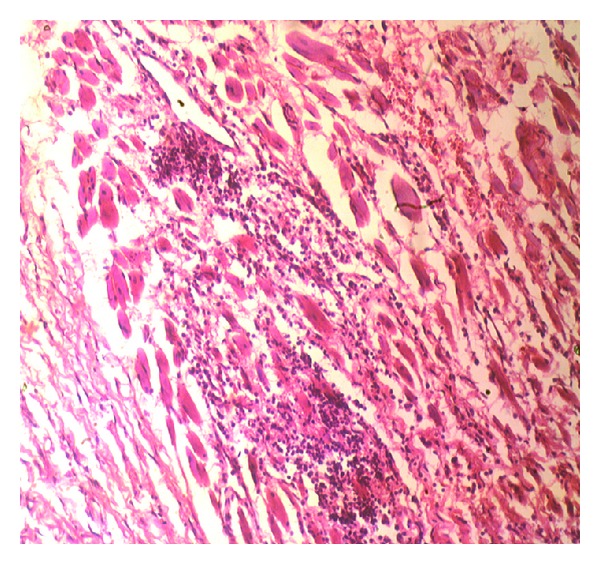
Histology slide of the frontal sinus soft-tissue mass. Note the spindled cells with wavy nuclei embedded in an acidophilic stroma (40x magnification).

 A diagnosis of neurofibroma was made on histopathological analysis (Figure 4). Of note, there were abundant spindle-shaped cells with wavy nuclei embedded in an acidophilic stroma. As well, some skeletal muscle (frontalis) infiltration with inflammatory cells was evident. 

**Figure 5:**
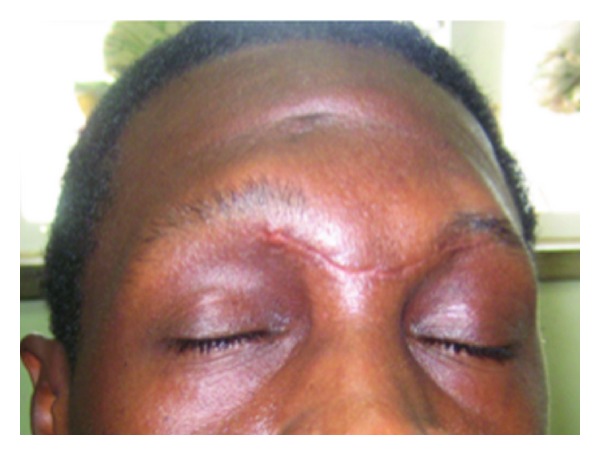
Postoperative photograph showing minimal deformity.

 Postoperatively, the incision healed well with minimal forehead scarring and deformity (Figure 5). No recurrence was noted with a 2-year followup.

## 3. Discussion

Neurofibromatosis can occur with multiple peripheral nerve-sheath tumors, which can at times involve the head and neck area [5]. Of these head and neck lesions, however, only 4 percent involve the paranasal sinuses and the nasal cavity [5]. Specifically, neurofibromas and schwannomas have been reported to occur in the nasal cavity [6], ethmoid sinus [7, 8], maxillary sinus [9, 10], and sphenoid sinus [11]. Interestingly, no solitary frontal sinus neurofibroma or schwannoma have been reported to date.

Neurofibromas are slow growing benign tumors, but can become very large causing compression of local structures, including expansion and erosion of adjacent bone [5]. In our case, the tumor seemed to have caused anterior frontal sinus wall erosion, which allowed the tumor growth to continue anteriorly and infiltrate the frontalis muscle in the forehead.

Clinically, neurofibromas present like any other benign lesions and the presenting signs and symptoms depend on the site of the tumor and subsequent involvement of surrounding structures [5]. In our case the only presenting complaint was a forehead swelling.

Imaging for these lesions include CT scanning and magnetic resonance imaging (MRI) to demonstrate the extent of the tumor, which is vital for planning of surgical excision. In most cases, MRI is thought to be superior to CT images since the former can allow better differentiation of the tumor from adjacent soft tissues, as well as improved evaluation of possible intracranial and intraorbital extension [5, 11]. Unfortunately, MRI is not available at the lead author's institution and therefore only CT scan was performed in our case.

The differential diagnosis of benign nasal cavity and paranasal sinus tumors mainly include fibroma, neurofibroma, papilloma, leiomyoma, and schwannoma [5, 12]. Histopathologic examination confirmed the diagnosis of neurofibroma in our case. 

The overall management of neurofibromas depends on the presenting signs and symptoms. Some lesions that are small and not causing any issues may not require any intervention, other than monitoring. As mentioned above, with growth however, there may be compressive symptoms, such as pain, cosmetic deformity, and neurovascular compromise. As well, there may also be a suspicion of the tumor being malignant, which necessitates surgical exploration, biopsy, and excision [5, 6].

Hemorrhage may occur during the operative intervention for neurofibromas due to the rich vascular stroma [5] but this was not observed in our case. Histopathologic analysis usually reveals a lesion with a lack of encapsulation and many elongated or spindle-shaped cells with oval or flattened nuclei [6, 12], which are all features noted in our specimen.

Recurrences have been reported to be infrequent although it is mentioned in the literature that tumors with locally invasive tendencies may recur after removal [5, 12]. With a 2-year follow-up, no recurrence was noted for the present case.

## 4. Conclusion

An isolated neurofibroma of the frontal sinus in a young male patient is presented. The tumor was treated successfully with surgical excision. To the authors' knowledge, no similar case has been reported.
